# Pooling of Patient-Derived Mesenchymal Stromal Cells Reduces Inter-Individual Confounder-Associated Variation without Negative Impact on Cell Viability, Proliferation and Osteogenic Differentiation

**DOI:** 10.3390/cells8060633

**Published:** 2019-06-24

**Authors:** Benedikt Widholz, Stefanos Tsitlakidis, Bruno Reible, Arash Moghaddam, Fabian Westhauser

**Affiliations:** 1 Center of Orthopedics, Traumatology, and Spinal Cord Injury, Heidelberg University Hospital, Schlierbacher Landstraße 200a, 69118 Heidelberg, Germany; benedikt.widholz@med.uni-heidelberg.de (B.W.); stefanos.tsitlakidis@med.uni-heidelberg.de (S.T.); bruno.reible@gmail.com (B.R.); Arash.Moghaddam-Alvandi@klinikum-ab-alz.de (A.M.); 2 ATORG - Aschaffenburg Trauma and Orthopedic Research Group, Center for Trauma Surgery, Orthopedics, and Sports Medicine, Klinikum Aschaffenburg-Alzenau, Am Hasenkopf 1, 63739 Aschaffenburg, Germany

**Keywords:** human mesenchymal stromal cells, donor-specific variability, osteogenic differentiation, bone tissue engineering, cell pooling, cell proliferation

## Abstract

Patient-derived mesenchymal stromal cells (MSCs) play a key role in bone tissue engineering. Various donor-specific factors were identified causing significant variability in the biological properties of MSCs impairing quality of data and inter-study comparability. These limitations might be overcome by pooling cells of different donors. However, the effects of pooling on osteogenic differentiation, proliferation and vitality remain unknown and have, therefore, been evaluated in this study. MSCs of 10 donors were cultivated and differentiated into osteogenic lineage individually and in a pooled setting, containing MSCs of each donor in equal parts. Proliferation was evaluated in expansion (assessment of generation time) and differentiation (quantification of dsDNA content) conditions. Vitality was visualized by a fluorescence-microscopy-based live/dead assay. Osteogenic differentiation was assessed by quantification of alkaline phosphatase (ALP) activity and extracellular calcium deposition. Compared to the individual setting, generation time of pooled MSCs was shorter and proliferation was increased during differentiation with significantly lower variances. Calcium deposition was comparable, while variances were significantly higher in the individual setting. ALP activity showed high variance in both groups, but increased comparably during the incubation period. In conclusion, MSC pooling helps to compensate donor-dependent variability and does not negatively influence MSC vitality, proliferation and osteogenic differentiation.

## 1. Introduction

Bone tissue engineering (BTE) belongs to the most dynamic research fields in basic science and involves different disciplines [1]. One of the major fields in BTE is the development and characterization of synthetic bone substitutes that might reduce or even replace the application of autologous bone in bone defect treatment, since autologous bone grafting can be associated with complications [2,3]. The respective experimental protocols, e.g., for the biological evaluation of synthetic bone substitutes, are based on the use of precursor cells that have the ability to develop and/or differentiate towards osteogenic lineage [4,5]. The use of standardized and well-described cell lines such as the MG63 osteoblast-like cells provides improved inter-study comparability, however, these (tumor) cell lines do differ significantly from actual osteoblasts or “wild-type” bone precursor cells [6].

An attractive alternative to the use of cell lines within BTE protocols are precursor cells such as mesenchymal stromal cells (MSCs) that can be isolated from donor patients and introduced to experimental settings [7,8]. However, donor specific variabilities concerning cell proliferation rates, osteogenic differentiation and cell vitality were shown to limit inter-study comparability, resulting in impaired data quality due to significant variances in the cell behavior [9,10,11]. Many factors such as donor’s age, sex, hormone status, diseases such as diabetes or obesity, or the abuse of substances like nicotine and alcohol are causing the inter-individual variability via complex mechanisms [12,13,14,15,16,17,18,19,20,21,22].

A possible approach to improve and standardize culture conditions in BTE experiments could be the pooling of human MSC from different donors in order to diminish inter-individual differences in cell proliferation, cell vitality and osteogenic differentiation. This setting has already been used in the past [23,24,25] and might allow the use of patient-derived cells with decreased cellular variability for a more stable application in BTE protocols. However, to our knowledge, there is insufficient evidence on the effects and influence of cell pooling, especially on osteogenic differentiation, proliferation and cell viability. This means that, so far, the effects of MSC pooling on the described biological properties of cell populations are unknown.

In this context, the objective of this study was to analyze and compare the influence of cell pooling of human MSCs on cell proliferation, cell viability and osteogenic differentiation using well-established methods and protocols. Furthermore, the impact of cell pooling on the data variances caused by inter-individual differences was evaluated. For that, MSCs of 10 donors were cultivated individually and in a pooled setting containing MSCs of each donor in equal parts. Cell proliferation was assessed via the calculation of generation times during expansion and quantification of dsDNA amount during osteogenic differentiation. Furthermore, cell viability and growth patterns were visualized by a fluorescence-microcopy-based live/dead assay. Osteogenic differentiation was quantified by determination of alkaline phosphatase activity (ALP) as a marker enzyme of osteoblasts and by evaluation of extracellular calcium depositions stained with Alizarin Red.

## 2. Materials and Methods

### 2.1. Study Overview

**Figure 1:**
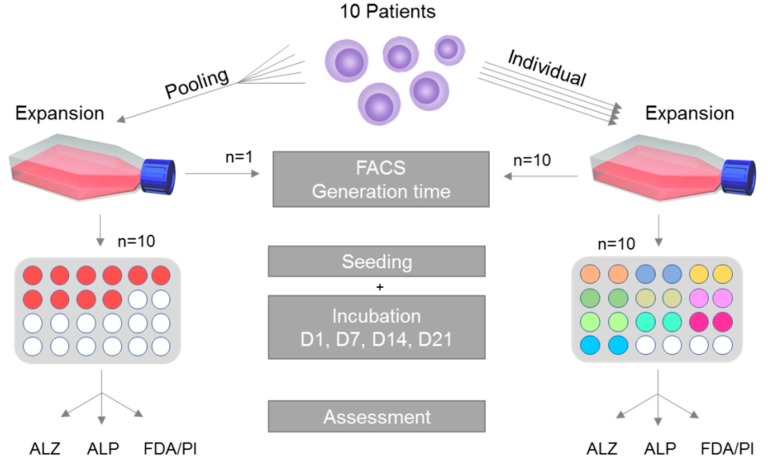
Overview of the experimental setup. Mesenchymal stromal cells (MSCs) of 10 patients were harvested and isolated. Next, the cells were expanded to passage 3 in cell culture flasks, and then seeded on 24 well plates. Ten replicates for the individual setting, consisting of 2 technical replicates each, and 10 replicates for the pooled setting. After designated incubation times, Alizarin Red S staining (ALZ), alkaline phosphatase activity measurement (ALP) and fluorescein diacetate (FDA)/propidium iodide (PI) staining (FDA/PI) were performed.

A schematic overview of the study is given in Figure 1. In short, MSCs are harvested from 10 patients and isolated following standardized and well-established protocols [5,26,27]. Cells of each patient in passage 1 were cultivated individually in addition to MSCs of all patients being pooled in equal parts in a single reaction tube and cultivated in the pooled setting. After expansion until passage 3, the generation time, the ability for trilineage differentiation as well as the MSC-specific surface characteristics were assessed during cultivation. After that, cells were subjected to osteogenic differentiation. The respective methods are explained in greater detail within the following paragraphs.

### 2.2. Study Ethics, Demography of Donor Patients and Primary Cell Culture

Table 1 provides a detailed overview of the donor patient characteristics. The collective of donors was composed of 6 men and 4 women with an average age of 49.7 (range 29.4–73.4, median: 45.7) years and an average body mass index (BMI) of 32.5 (range 22.4–41.6; median: 33.1) kg/m^2^ that underwent surgery at the proximal femur for medical reasons at the Heidelberg Orthopedic University Hospital. Written consent was obtained prior to collection of the material. The responsible ethics committee of the Medical Faculty of the University of Heidelberg approved the study (S-443/2015). The donor patients were selected randomly.

Isolation of MSCs was conducted as published previously [5]. In short, bone marrow was washed with phosphate buffered saline (PBS) (Life Technologies, Darmstadt, Germany) and transferred to 0.1% gelatin-coated (Sigma-Aldrich, Steinheim, Germany) T75 flasks (Nunc, Roskilde, Denmark). Cultivation was performed in expansion medium (83% Dulbecco’s modified Eagle’s medium (DMEM) high glucose, no glutamine, 14% fetal calf serum (FCS), 2 mM l-glutamine, 1% non-essential amino acids (NEAA), 50 µM ß-mercaptoethanol (all Thermo Fisher, Dreieich, Germany), 100 µg/mL penicillin/streptomycin, 2.5 µg/mL amphotericin B (both Biochrom, Berlin, Germany), 4 ng/mL fibroblast growth factor 2 (Abcam, Berlin, Germany). The medium was changed after 24 h to remove any non-adherent cells and incubation was proceeded with for 10 days. The tissue material was then removed, and the adherent cells were transferred to the next passage in new T75 flasks. Repetitive passaging was conducted when the cells reached a confluence of 80%. Media changes were performed twice per week.

### 2.3. Single Cultivation, Pooled Cultivation and Generation Time

Cells in passage 1 were subjected to the two experimental settings: (i) MSCs of each patient were cultivated individually or (ii) MSCs of all patients were pooled in equal parts in a single reaction tube and cultivated in the pooled setting; 450,000 cells for each approach were seeded in T75 flasks and then expanded.

The generation time of the cell populations in expansion conditions was assessed between splitting to passage 3 and transferring the cells to the osteogenic differentiation setting as described below. The time between cell seeding and 80% confluency was noted. After reaching 80% confluency, cells were detached and counted with a hemocytometer. The generation time was calculated using the formula [28]:

G = (log2 × T)/(logN1 − logN0)(1)

G = generation time; T = incubation time [hours]; N0 = cell count at seeding time point; N1 = cell count at harvesting time point.

One cell culture flask each was used per patient in the individual setting and one for the pooled setting. Therefore, analysis of the generation time was only performed individually and did not undergo statistical evaluation.

### 2.4. Cell Characterization

The isolated cell populations were characterized as MSCs according to consensus of the International Society for Cellular Therapy (ISCT) by their ability for trilineage (osteogenic, adipogenic, chondrogenic) differentiation, plastic adherence and specific surface antigen expression patterns analyzed by flow cytometry [29]. Plastic adherence of the cells was demonstrated during cell expansion. There is a separate chapter for analysis of osteogenic differentiation since this is one of the main objectives of this study. Characterization was performed for both the individual and the pooled culture setting.

#### 2.4.1. Flow Cytometry

According to the criteria set by the ISCT, >95% of the cells must be positive for CD105, CD73 and CD90 and <2% must be positive for CD14, CD20, CD34 and CD45. To verify this, an MSC Phenotyping Kit (Miltenyi Biotech, Bergisch Gladbach, Germany) was used following the manufacturer’s instructions and samples were analyzed with a MACSQuant^®^ Analyzer 10 (Miltenyi Biotech, Bergisch Gladbach, Germany). One sample was analyzed for each donor in the individual setting and one sample in total for the pooled setting.

#### 2.4.2. Adipogenic Differentiation

**Figure 2:**
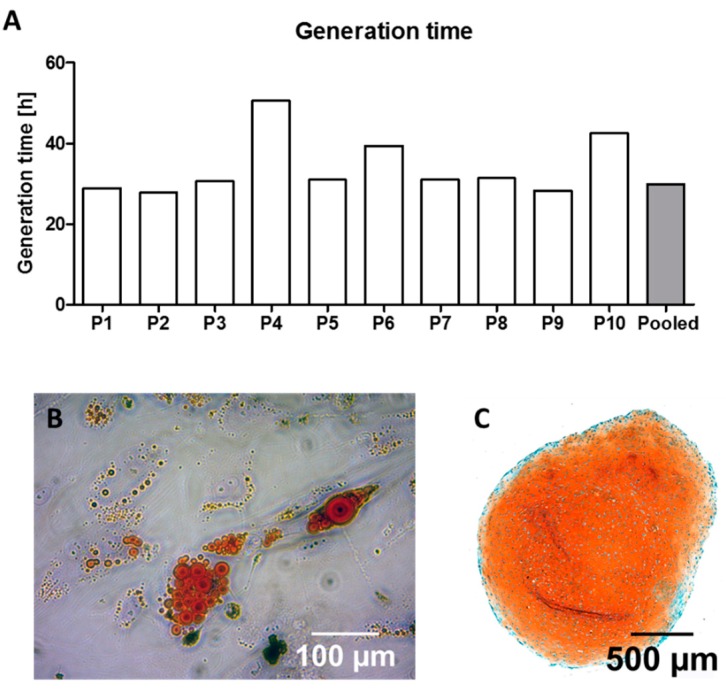
Generation time and characterization of donor cells as MSC. Generation time of 10 single patients (P1–P10) and pooled cells (**A**). Representative pictures of red-colored fatty vacuoles after adipogenic differentiation for the pooled group after Oilred-O staining (**B**) and orange staining indicating chondrogenic differentiation for the pooled group in Safranin-O/Fast Green staining (**C**).

For 14 days, 35,000 MSC were cultivated in adipogenic induction medium (87.8% DMEM high glucose, 8.8% FCS, 4 ng/mL insulin glargine (Sanofi-Aventis, Frankfurt am Main, Germany) 90 µg/mL penicillin/streptomycin, 2.2 µg/mL amphotericin B, 0.05 mM isobutyl methylxanthine, 1 µM dexamethasone (all Sigma-Aldrich, Steinheim, Germany), followed by Oilred-O staining and counterstaining with haemalaun solution (both Waldeck, Muenster, Germany). To confirm adipogenic differentiation, the presence of red-stained fatty vacuoles was examined microscopically. One sample was analyzed for each donor in the individual setting and one sample in total for the pooled setting. Results are shown representatively (Figure 2).

#### 2.4.3. Chondrogenic Differentiation

For chondrogenic differentiation, 500,000 MSCs were centrifuged for 5 min at 800× *g* into a pellet and then incubated in chondrogenic induction medium (94% DMEM high glucose, 40 µg/mL transferrin, 40 µg/mL sodium selenite, 1 µM dexamethasone, 0.17 mM ascorbic acid 2-phosphate, 1 mM sodium pyruvate, 0.35 mM proline, 1.25 mg/mL bovine serum albumin (all Sigma-Aldrich, Steinheim, Germany), 100 µg/mL penicillin/streptomycin, 2.2 µg/mL amphotericin B, 0.1375 IE/mL insulin glargine (Sanofi-Aventis, Frankfurt am Main, Germany), and 10 ng/mL transforming growth factor β1 (Abcam, Berlin, Germany) for 42 days. Afterwards the pellets were fixed for 2 h in 4% paraformaldehyde (Merck, Darmstadt, Germany) and then dehydrated for 2 h in 70%, 96% and 100% 2-propanol, followed by a 30 min incubation in 100% acetone (all Carl Roth, Karlsruhe, Germany). The pellets were then transferred into paraffin and processed into sections for histological evaluation by Safranin-O/Fast Green (Waldeck, Muenster, Germany) staining. A qualitative analysis for orange stained proteoglycans and glycosaminoglycans as a marker for the development of cartilage tissue was microscopically conducted. One sample was analyzed for each donor in the individual setting and one sample in total for the pooled setting. Results are shown representatively (Figure 2).

### 2.5. Osteogenic Differentiation: General Culture Setting

To evaluate the osteogenic potential, 35,000 MSCs per well were transferred into 24-well plates (Nunc, Rosklide, Denmark) and cultured in osteogenic differentiation medium (86% DMEM high-glucose, 10% FCS, 100 µg/mL penicillin/streptomycin, 2.5 µg/mL amphotericin B, 0.1 µM dexamethasone (Sigma Aldrich, Steinheim, Germany), 2.5 µg/mL ascorbic acid-2-phosphate (Sigma-Aldrich, Steinheim, Germany), 10 mM beta glycerophosphate (Merck, Darmstadt, Germany).

For the individual setting, MSCs of each donor were seeded in duplicates. In the pooled setting 10 replicates were cultured. Quantification of osteogenic differentiation was performed on day 1 (D1), 7 (D7), 14 (D14) and 21 (D21). Media were changed twice per week.

### 2.6. Osteogenic Differentiation: Quantification of Alkaline Phosphatase (ALP) Activity

ALP converts para-nitrophenylphosphate (p-NPP) to para-nitrophenol (p-NP). The conversion correlates with the ALP activity in the sample and the change of color in the solution from transparent to yellow can be measured spectrometrically [26,27]. ALP assessment was performed as published previously [26,27]. In short, MSCs were lysed with 1% Triton X-100 (Sigma-Aldrich, Steinheim, Germany) and subjected to ALP buffer (0.1 M glycine, 1 mM MgCl_2_, 1 mM ZnCl_2_, pH 10.4). After 90 min, the change in color was measured at 405/490 nm in a Dynatech MLX microplate reader (Dynatech Laboratories, Stuttgart, Germany). To normalize the results to variances in cell quantity, the amount of total protein in each sample was determined by conducting a Micro BCA Protein Assay (Thermo Fisher, Dreieich, Germany) according to the manufacturer’s instructions. All samples were measured as technical duplicates.

### 2.7. Osteogenic Differentiation: Quantification of Extracellular Calcium Depositions

To quantify the amount of extracellular calcium deposition, the cells were subjected to Alizarin Red S staining as published previously [26,27]. In short, cells were fixed in 70% ethanol (Carl Roth, Karlsruhe, Germany), incubated over night at 4 °C, washed with Aqua dest. and then stained with 0.5% Alizarin Red S solution (Waldeck, Münster, Germany) for 10 min. After washing with PBS, a 10% hexadecylpyridinium chloride solution (Merck, Darmstadt, Germany) was added to each sample and incubated on an oscillator (IKA-Werke, Staufen, Germany) for 30 min at 350 rpm to dissolve the stained calcium depositions. After complete dissolution each sample was measured spectrometrically at 570 nm as technical duplicates and normalized to a standard curve.

### 2.8. Osteogenic Differentiation: Evaluation of Cell Proliferation, Growth Patterns and Viability

The amount of dsDNA, correlating with the number of cells per sample, was determined using the Quant-IT PicoGreen dsDNA Assay Kit (Thermo Fisher Scientific, Dreieich, Germany) according to the manufacturer’s instructions. In summary, cells underwent lysis in 1% Triton X-100, before the lysates were diluted 1:10 in Tris-EDTA Buffer (200 mM Tris-HCl, 20 mM EDTA, pH 7.5). Then the PicoGreen staining solution was added and incubated for 3 min at room temperature protected from light, before fluorescence was measured in a Wallac Victor 2 (Perkin Elmer, Waltham, MA).

Cell viability and growth patterns were qualitatively visualized by a fluorescence-microcopy-based live/dead assay. First, cells were washed with PBS, before a staining solution (2 µg/mL fluorescein diacetate (FDA), 20 µg/ml propidium iodide (PI) (both Thermo Fisher, Dreieich, Germany)) was added and incubated in darkness for 5 min at room temperature. The staining solution was then discarded, followed by another washing step with PBS. Afterwards each sample was examined and photographed with an Olympus IX81 Microscope (Olympus, Tokyo, Japan). FDA binds to the cell membrane of living cells, dyeing it green [5]. In contrast, PI cannot penetrate the cell membrane but binds to free DNA released from dead cells, which can be detected as red color [5].

### 2.9. Statistics

The statistical analysis was performed with IBM SPSS Statistics 25 (IBM, Armonk, NY). A two-sided *t*-test was applied in which *p*-values of <0.05 were regarded as significant. To determine significant differences regarding variances among groups, Levene’s test was applied for all groups and timepoints. For the quantitative experiments, *n* = 10 replicates were used for the pooled setting. Cells of each patient were cultivated in duplicates within the individual setting. Average values (one per patient, resulting in *n* = 10 in total) were used for comparison to the pooled group. The generation time was not assessed statistically, as only one cell culture flask was expanded per patient and the pooled setting. Values are shown as rounded means. Variances are shown as standard deviation where applicable. Graphs were created using GraphPad Prism (Version 5.01; GraphPad Software, La Jolla, CA, USA).

## 3. Results

### 3.1. Patient Collective

The patient collective that has been randomly selected for this study showed a heterogeneous character (Table 1). Patients without relevant confounders (e.g., P9) were part of the collective as well as patients with a major risk profile that could potentially influence the MSC behavior (e.g., P7). Considering the World Health Organization (WHO) BMI classification, the majority of the patients (8 out of 10) were overweight with a BMI ≥ 25 kg/m^2^ [30].

### 3.2. Generation Time and Cell Characteristics under Expansion Conditions

In order to analyze whether cell pooling affects the growth rate of the cells under expansion conditions, generation time was calculated by analyzing the time necessary for the cell population to double. These timeframes showed a broad inter-individual variance among the donors (34.14 ± 5.53 h). In the pooled setting the generation time was shorter (29.81 h) compared to the average time of the individual settings (Figure 2A).

After expansion, the cell populations had to be defined as MSC according to characteristics defined by consensus of the ISCT [29]. During cell cultivation, plastic adherence was observed in all groups. Furthermore, all cell populations in both settings were able to differentiate into adipogenic and chondrogenic lineage (Figure 2) and exhibited the characteristic surface receptor expression patterns (Table 2). The definition of trilineage differentiation was performed once per patient (resulting in 10 replicate values) and once for the pooled sample (resulting in one replicate value).

### 3.3. Extracellular Calcium Deposition

The extracellular deposition of calcium positively correlates with the osteogenic differentiation of a cell population [26,27]. Therefore, calcium depositions were visualized in representative samples and quantified for the whole experimental setting.

**Figure 3:**
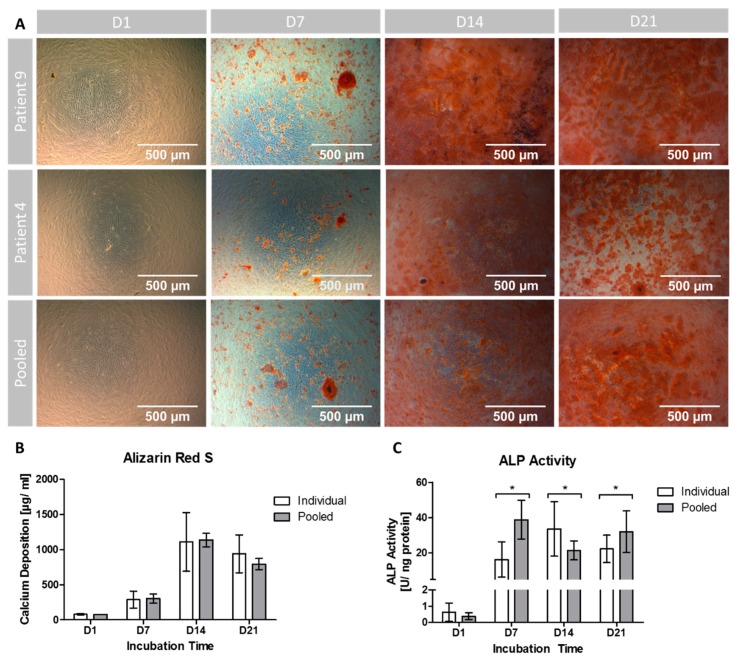
Evaluation of osteogenic differentiation. (**A**) Alizarin Red S stained extracellular calcium deposition of 2 representative patients (P4 and P9, respectively) and the pooled group over the cultivation period of 21 days. (**B**) Quantification of extracellular calcium deposition. (**C**) Quantification of ALP activity over the incubation period. (*) indicates significant differences. Individual: Data obtained from 10 patients (*n* = 2 technical replicates for each donor). Pooled: Data obtained from *n* = 10 replicates.

To visualize potential differences in the ability to generate extracellular calcium depositions among individuals, patient 9 (P9) with a short and patient 4 (P4) with a long generation time were chosen as representative samples for microscopic evaluation and compared to the pooled setting. As seen in Figure 3A, no calcium patches were stained on D1 in all samples. On D7 single red stained calcium depositions had formed in all groups, distributed as patches among the cell layer. On D14 dense accumulations of calcium appeared with a maximum in the sample of P9. On D21, P9 and P4 displayed a slight decrease in staining, while the pooled setting showed a minor increase of red stained patches.

Quantitatively, the amount of extracellular calcium deposition was non-significantly (*p* = 0.222) lower on D1 in the pooled setting compared to the individual cultivation setting (Figure 3B). On D7 (*p* = 0.716) as well as on D14 (*p* = 0.835) the pooled setting showed non-significantly higher calcium levels compared to the individual setting. The peak calcium deposition was reached on D14 in both groups (with an absolute maximum in the pooled group) and dropped until D21. On D21 higher calcium deposition levels in the individual setting compared to the pooled group were found, but again no significance was reached (*p* = 0.129).

During all observation time points, variances were higher in the individual setting. These differences were significant with exception for D7 (D1: *p* = 0.002; D7: *p* = 0.104; D14: *p* = 0.001; D21: *p* = 0.008).

### 3.4. ALP Activity

ALP is a marker enzyme for osteoblasts and was therefore quantified as another representative parameter for osteogenic differentiation of the precursor cell population.

ALP activity levels started on D1 at approximately the same values without significant differences between the cultivation settings (*p* = 0.214; Figure 3C). On D7, the pooled setting reached the overall maximum in ALP activity with significant differences to the individual setting (*p* < 0.001). The ALP activity in the individual setting peaked on D14 and was significantly higher (*p* = 0.039) compared to the pooled setting. On D21, the activity levels declined in the individual setting, whereas in the pooled setting ALP activity increased again to form a second peak, resulting in significant (*p* = 0.043) differences. However, during the incubation time both groups showed an increase in ALP activity being a correlate of ongoing osteogenic differentiation.

The variances were higher in the individual setting on D1 and D14 and higher in the pooled setting on D7 and D21. However, only on D14 the differences in variances were significant (*p* < 0.001).

### 3.5. Cell Proliferation, Growth Patterns and Viability during Osteogenic Differentiation

**Figure 4:**
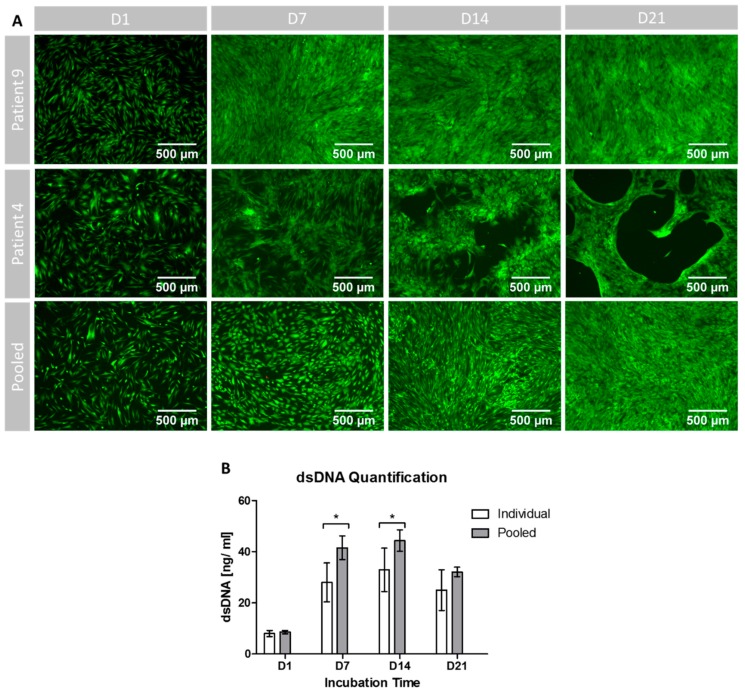
Proliferation and cell survival under osteogenic differentiation conditions. (**A**) Visualization of viability and cell growth patterns during the incubation period of 21 days for 2 representative single patients (P4: slow growth during expansion; P9: fast growth during expansion) and the pooled setting. (**B**) Amount of dsDNA per sample during the incubation time. (*) indicates significant differences. Individual: data obtained from 10 patients (*n* = 2 technical replicates for each donor). Pooled: data obtained from *n* = 10 replicates.

The differences in generation time and cell growth that had already been detected during cell expansion also continued under osteogenic differentiation settings (Figure 4A). Starting with a comparable amount and growth pattern of the cells on D1, the MSCs of P9 displayed confluent growth patterns already on D7, whilst the cells of P4 and the cells within the pooled setting were not confluent yet. On D14, cells in the pooled setting reached confluence as well, while the cells of P4 developed distinct free spots in the cell layer that developed into cell free circular holes on D21. From D14 on, the pooled group and P9 showed confluent growth patterns with increasing density towards D21.

As shown in Figure 4A, the MSC were mostly stained green, indicating viable cells. In addition, only very few red PI-dyed DNA molecules were found in each sample. Therefore, in qualitative analysis, the cell survival was not impaired by pooling and equally high for all groups at all timepoints.

The quantification of dsDNA count confirmed the qualitative findings of inter-individual differences: the variances were higher at all time points for the individual setting, even with significant differences on D21 (*p* = 0.003; Figure 4B).

The pooled setting showed higher dsDNA levels than the individual setting at all time points (Figure 3B). Except for D1 (*p* = 0.228), these differences were significant (D7: *p* < 0.001; D14: *p* = 0.002; D21: *p* = 0.020). In both settings the highest dsDNA content was detected on D14 and then declined until D21.

## 4. Discussion

Human MSCs play a key role in BTE and especially in the evaluation of new synthetic bone substitutes. However, the use of MSCs in basic experimental orthopedic research is limited by their known donor-dependent variabilities concerning cell proliferation, osteogenic differentiation and cell viability, reducing inter-study comparability [9,10,11]. Furthermore, studies have to be conducted using several patient-derived cell lines in order to compensate for donor-caused variations [9]. Various donor specific factors such as age [14,21], sex [12,15], alcohol and/or tobacco consumption [19,22,31], and morbidities such as obesity or diabetes [17,20] have been identified in the past to cause this inter-individual variability via complex mechanisms [16,18]. Inter alia, Hong et al. have shown that osteogenic differentiation and cell proliferation are influenced by intrinsic steroid regulation causing considerable sex-linked variability [15]. In their basic experimental research, investigating the influence of obesity and metabolic syndrome on osteogenic differentiation, Oliva-Olivera et al. could show that the osteogenic potential of omental adipose-derived cells is decreased considerably in patients with a body mass index greater than 25 kg/m^2^ and the presence of metabolic syndrome [17]. In addition, age and exposure to cigarette smoke are affecting MSC viability as well as their differentiation patterns by emphasizing adipogenic respectively affecting osteogenic differentiation [13,19].

In order to limit these influences and variances, MSCs of different donors can be pooled—this approach has already been used in the past [23,24,25]. To our knowledge, there is a lack of data analyzing whether cell proliferation, viability and osteogenic differentiation is influenced by cell pooling. Therefore, the objective of this in-vitro study was to analyze the effects of cell pooling on human MSCs regarding variability of osteogenic differentiation, cell survival and proliferation rates.

The patient collective that has been randomly selected for this study showed heterogeneous character, including patients with and without relevant confounders that influence MSC behavior, making this collective suitable to evaluate individual differences and a possible way to overcome these by cell pooling. However, it must be considered that 80% of the patients were overweight. Therefore, it might be assumed that the osteogenic performance of the majority of the cells was lower compared to cells being isolated from a collective of patients with normal weight [17]. Based on differences in the generation time, two donors were selected for representative staining of extracellular calcium deposition as well as for qualitative fluorescence-microcopy-guided growth pattern and vitality analysis. P4 showed the slowest generation time during expansion and was with 74 years the oldest patient within the collective. Also, the BMI of P4 (35.1 kg/m^2^) was slightly higher than the average BMI (32.5 kg/m^2^). Whilst P4 was female, P9 was a male patient who was younger compared to the average age of the collective and showed fast generation times (28.28 h). Interestingly, P9 had the lowest BMI (22.4 kg/m^2^) amongst the collective. Based on these two representative donors, it seems that age, sex and weight have a potent influence on cell proliferation during expansion and osteogenic differentiation even within the selected collective [32].

During cell expansion, inter-individual differences between generation times were also quantifiable: the fastest cell population (P2) doubled within 27.76 h whilst it took the slowest cell population (P4) 50.59 h to double (Figure 2A). These findings, describing the inter-individual variances, are coherent with the findings of previously conducted studies that analyzed the proliferation rates of MSC using comparable protocols [33,34,35]. Compared to the pooled population that showed a doubling time of 29.81 h, the average doubling time of the individual cell culture settings was higher with 34.14 h (±7.53 h). This might lead to the assumption that the fast proliferating cells contribute more towards the whole cell population in the pooled setting, resulting in faster overall proliferation rate and eventually increasing the relative portion of the respective cells. For the differentiation experiments, the proposed context could be of certain relevance: it has been shown for chondrogenic differentiation of MSC that proliferation rates and differentiation capacity are closely related [35,36]. For osteogenic differentiation, it was demonstrated that seeding density and confluence of MSCs has an impact on osteogenic differentiation; higher density and confluence (around 80%) leads to more pronounced osteogenic differentiation [37,38]. If the better proliferating cells would account for a larger proportion in the pooled setting after expansion, they might influence the osteogenic differentiation positively since they would reach confluence in the differentiation setting faster.

The qualitative assessment of cell viability by FDA/PI staining did not show differences in the green fluorescent intensity between the pooled and the individual setting (Figure 4A). Interestingly, the growth patterns of the slowly proliferating cells of patient P4 differed from those of the pooled cells and of the faster proliferating cells of patient P9. The cell layer appeared to show bald spots in P4 whilst the growth patterns were more confluent in the two other settings. However, despite the different growth patterns, viability seemed to be the same for all settings. The used FDA/PI staining is established to determine cell viability in different cell culture settings, however, within the used protocol it remains on the low evidence level of qualitative results [39]. Furthermore, more detailed methods will allow us to understand changes in cell vitality to a greater extent, e.g., Annexin/PI staining for the monitoring of apoptosis.

Different approaches were used to analyze the osteogenic differentiation of the MSC population. Extracellular calcium deposition was qualitatively visualized and quantified by Alizarin Red S staining. Whilst both groups showed increasing calcium depositions over time from D1 to D14, the variances were significantly higher in the group of individuals compared to the pooled setting. The absolute concentration of extracellular calcium was comparable, although no significant differences were found when comparing both cultivation settings. It might therefore be concluded that the pooled setting represents—in terms of extracellular calcium deposition—the average osteogenic differentiation potential of the single donors with significantly lower variances.

Regarding variance analysis it must be considered that the experimental approach of this study included only technical replicates in the pooled group. The variances in this group are therefore limited to technical and methodological issues. In the group of individuals however, the variances were caused by both technical and biological replicates since the MSCs of the patients have been analyzed in 10 individual settings. Therefore, the variances are expected to be lower in the pooled group since technical variances are assumed to be more or less equal/stable in both groups. The differences in variations between the two approaches can only be interpreted through careful consideration of the experimental setting. However, the used approach allows to determine whether the influence of biological variation is as strong as anticipated when directly compared to the technical variances [24,25]. We found that the additional “biological” variance causes significant differences in absolute variance between the individual and pooled group. This might therefore lead to the conclusion that the biological variances are of certain higher relevance compared to the influence of technical replicates, at least for the calcium deposition.

ALP activity increased during the incubation period as another correlate of early osteogenic differentiation in both groups [5,26,27]. However, ALP activity showed significant differences between the groups at various time points: on D7 and D21, significantly higher ALP activity was analyzed for the pooled setting. On D14, the individual culture setting showed significantly higher ALP activity. Interestingly, variances were higher in the individual group on D1 and D14 but lower compared to the pooled group on D7 and D21. However, only the difference at D14 was significant. These results seem to be contradictory at first, however some possible explanations can be found. For instance, as indicated above, the experimental setting used within this study allows us to evaluate the additional influence of biological variance. ALP activity is known to show variations in the used experimental setting because of its lower sensitivity for small ALP concentrations [40]. Indeed, within the presented data the variance was higher on D7 and D21 in the pooled group—these variances must have mainly been caused by technical reasons. Furthermore, ALP activity has to be normalized in order to exclude differences caused by differences or alterations in cell populations [26,27]. In this case, the protein content was used for normalization following established protocols [26,27]. Considering the fact that osteoblast precursor cells shift during differentiation from mainly ALP dominated protein production to the production of extracellular matrix proteins, the relative amount of ALP production is likely to decrease during differentiation and is prone to time-dependent variation [41]. Therefore, it might be of interest for further studies to evaluate the osteogenic differentiation of the cell population on a genetic level in order to cover all aspects of the osteogenic differentiation process: changes on a genetic level resulting in cellular and extracellular protein production and maturation e.g., via incorporation of calcium [41,42]. Like the quantity of extracellular calcium deposition, ALP activity also increased during the incubation time with maxima at D7 for the pooled setting and D14 for the individual group. However, while the maximum ALP activity for the pooled group was analyzed at D7, the maximum calcium deposition was measured at D14. For very similar culture settings, it has been described that the osteogenic differentiation takes place in three major phases [41,42]: the initial phase is mainly characterized by cell proliferation and takes place during the first four days in differentiation conditions [41,42]. During the second phase from day four to 14, an early differentiation of the precursor cell population takes place that is characterized by ALP production and generation of a primitive extracellular matrix [41,43]. The maturation of the extracellular matrix takes place during the last phase from day 14 onwards [41,42,44]. This means that ALP production happens earlier compared to the calcification of the extracellular matrix that can be considered as a maturation process. Since ALP activity peaks between D7 and D14, the maxima in both groups still fit into the proposed phase model [41,42]. Furthermore, calcium deposition as a marker of the maturation of the extracellular matrix shows similar patterns during the incubation period. It would be interesting to have other evaluation time points between D7 and D14 since – hypothetically – the maximum ALP expression can take place at any time during this period. Although ALP activity represents a marker for early osteogenic differentiation, the measurement was continued until D21 to examine whether the commonly observed decline of ALP activity could be observed in a pooled setting as well.

Since expansion and differentiation conditions have a relevant influence on cell behavior, cell proliferation was again assessed under differentiation conditions [37,38,45]. The donor-dependent differences in cell proliferation continued also under osteogenic differentiation conditions. The results from the expansion setting led to the assumption that the cells with better proliferation potential will contribute with a higher relative portion to the pooled population. This assumption is again supported by the findings under osteogenic differentiation conditions, showing significantly superior proliferation patterns on D7 and D14 for the pooled setting compared to the individual cultivation group that in addition showed higher variances, even with significant differences on D21. However, this conclusion can only be drawn for the proliferation patterns since the extracellular calcium deposits do not show significant differences between the two groups and the ALP activity assessment is prone to variations as discussed above. The differences in proliferation patterns are likely to have a certain influence on the cell population with increasing incubation and proliferation times: based on the assumption that the better proliferating cells will contribute more to the pooled cell population, they might influence the population stronger. This can lead to the problem that—after a certain amount of time—the “pooled” population will be more or less a population of the best growing single donor cells. Therefore, the characteristics of the cell population are likely to change or alter with increasing incubation time. However, since MSCs are known to change their properties with increasing passaging procedures, the influence of the different proliferation patterns might remain on a level of limited relevance: in a study recently conducted by Yang and coworkers, it was suggested to use MSCs in early passages for osteogenic differentiation experiments [46].

Further studies should focus on the evaluation of the osteogenic differentiation patterns in further detail, for example by gene expression analysis. Furthermore, genetic sequencing could be used to identify the individual contribution of each donor to the pooled cell population during expansion and differentiation. Based on the data obtained, the influence of the composition of the pool on the biological properties of the MSCs cannot be evaluated. For example, a different number of donor patients might result in an altered behavior of the MSCs. Future studies should assess the influence of different “pool compositions” e.g., regarding the number of patients included, on the biological properties of the pooled cell population.

## 5. Conclusions

In this study, we could demonstrate that that cell pooling does not negatively affect proliferation and osteogenic differentiation of human MSCs under in vitro conditions and supports compensation of donor-specific variability indicated by lower variances, thus improving quality of data. Despite slight alterations in the chronology of differentiation patterns, the osteogenic differentiation potential of both settings was comparable and variances were significantly reduced in the pooled setting. Our results suggest that cell pooling is suitable to reduce those confounder-associated effects in experimental BTE settings. Future studies should focus on the evaluation of the individual participation of each donor in the pooled setting and possible differences resulting from different pool compositions, e.g., by alteration of the number of donor patients. Further methods such as gene expression analysis might help us to understand the differences in chronology between the osteogenic differentiation patterns in greater detail.

## Figures and Tables

**Table 1:** Patient demographics.

Patient #	Age	Sex	BMI	Further Confounders
P1	69	W	22.7	None
P2	40	W	28.9	Analgesic abuse
P3	50	M	31.9	Cortisone therapy
P4	73	W	35.1	None
P5	29	M	41.6	None
P6	31	M	37.1	Cortisone therapy
P7	61	M	39.6	Metabolic syndrome, nicotine and alcohol abuse
P8	62	M	31.8	None
P9	39	M	22.4	None
P10	42	W	34.3	Nicotine abuse

Patient #: chronological numbering of the patients. Age rounded to full years. M: male; F: female. BMI: body mass index [kg/m^2^]. Further confounders: list of confounders other than weight and sex influencing MSC behavior.

**Table 2:** Evaluation of surface proteins for the pooled group necessary for MSC classification. Average with (±) standard deviation of the individual setting, composed of replicates of 10 patients. The pooled setting is represented by one replicate; therefore, no standard deviation is shown.

Group	CD105	CD73	CD90	Negative Control
Pooled	99.43%	96.59%	98.42%	1.14%
Individual	99.44 ± 0.14%	96.67 ± 1.60%	98.47 ± 0.77%	1.71 ± 0.24%
